# Air Tamponade for Rhegmatogenous Retinal Detachment With Inferior Breaks After 25-Gauge Pars Plana Vitrectomy: Technique and Outcome

**DOI:** 10.3389/fmed.2022.724234

**Published:** 2022-04-07

**Authors:** Peiyang Shen, Xiangbin Kong, Lijun Zhou, Peng Su, Xiaohe Lu, Mingguang He

**Affiliations:** ^1^Department of Ophthalmology, Zhujiang Hospital, Southern Medical University, Guangzhou, China; ^2^Department of Ophthalmology, The Second People’s Hospital of Foshan, Affiliated Foshan Hospital of Southern Medical University, Foshan, China; ^3^Department of Surgery, Centre for Eye Research Australia, Ophthalmology, University of Melbourne, Melbourne, VIC, Australia; ^4^State Key Laboratory of Ophthalmology, Zhongshan Ophthalmic Center, Sun Yat-sen University, Guangzhou, China

**Keywords:** air tamponade, inferior breaks, 25-gauge, pars plana vitrectomy, rhegmatogenous retinal detachment

## Abstract

To evaluate the outcomes of 25-guage (G) pars plana vitrectomy (PPV) with air tamponade for rhegmatogenous retinal detachment (RRD) with inferior breaks. This retrospective consecutive case series included fifty-two eyes of fifty-two RRD patients with inferior breaks who underwent 25-G PPV with air tamponade. These patients were followed up for at least 6 months following surgery. Primary and final anatomical success rates and postoperative complications were the main outcome measures. The mean age of the patients (39 men and 13 women) was 51.8 ± 11.8 years. There were 49 primary RRDs (94.2%) and three recurrent RRDs (5.8%). The mean follow-up period was 8.2 ± 1.6 months (range: 6–13 months). Sixteen eyes (30.8%) presented with high myopia, and six eyes (11.5%) were pseudophakic. Proliferative vitreous retinopathy grade was C1 in four eyes (7.7%). Of the 52 eyes, two (3.8%) were complicated with choroidal detachment, and forty (76.9%) had the macula detached. The single- and final-operation success rates were 96.2% and 100%, respectively. During follow-up, secondary cataract surgery was performed in eight eyes (17.4%) of the 46 phakic eyes. 25-G PPV with air tamponade is effective in treating selected RRD patients with inferior breaks. Patients can benefit from early visual recovery and less complications.

## Introduction

Rhegmatogenous retinal detachment (RRD) is a severe vision-threatening disease, defined as the separation of the inner neurosensory retina and the outer retinal pigment epithelium (RPE) due to retinal breaks formation caused by abnormal vitreoretinal traction (1). At present, pars plana vitrectomy (PPV) gains popularity in treating RRD due to its low invasiveness and adequate visualization of the peripheral retina via the wide-angle viewing system (2–5). After removing the vitreous, long-acting gas, such as 14% perfluoropropane (C_3_F_8_) and 18% sulfur hexafluoride (SF_6_), or silicone oil are injected into the vitreous cavity to support retinal breaks closure and assure chorioretinal adhesion establishment.

Since April 2016, long-acting gases were no longer commercially available in China because of legislation changes. Silicone oil carries potential complications such as elevated intraocular pressure (IOP) and the necessity of secondary removal surgery (6). The current preference includes using air as a reasonable alternative for the tamponade (5, 7, 8). The air has a shorter half-life and a lower risk of elevated IOP and cataract development or progression compared to other agents. Previous studies have suggested that air can perform on par with long-acting gas in terms of surgical outcome (5, 9). However, air tamponade is generally indicated for RRDs with superior breaks; yet, the inferior breaks always pose a challenge for air tamponade (10–13). Therefore, we conduct this retrospective study to evaluate the surgical outcomes of RRD patients with inferior retinal breaks treated with 25-gauge (G) PPV using air tamponade only.

## Materials and Methods

We reviewed medical records of all RRD patients from April 2018 to April 2021 who underwent 25-G PPV with air tamponade. These patients had at least one causative break located in the inferior retina (between 4 and 8 o’clock meridian). The exclusion criteria included giant retinal tears, proliferative vitreous retinopathy (PVR) grade C2 or greater, myopic macular hole-associated retinal detachments, traumatic RRDs, pediatric RRDs (birth to 18 years of age), other serious eye diseases, and less than 6 months of follow-up. The Ethics Committee of the Second People’s Hospital of Foshan approved the study. It was performed following the Tenets of the World Medical Association’s Declaration of Helsinki. Written consent for surgical treatment was obtained from each patient.

All eligible patients underwent comprehensive ophthalmic examinations, including Snellen best-corrected visual acuity (BCVA) measurement, non-contact tonometry, fundus examination, B-ultrasonography, and optical coherence tomography (OCT). The characteristics (number, size, type, and location) of retinal breaks and other procedures details (e.g., combined lens extraction and drainage retinotomy) were extracted from surgical records. PVR diagnosis was made according to the classification of the American Retina Society Terminology Committee, United States (14).

Two surgeons conducted all surgeries under retrobulbar anesthesia (PSh and XK). After proper conjunctival sac disinfection using povidone-iodine, a standard three-port 25-G PPV was performed using the Alcon Constellation Vision System (Alcon Laboratories, Fort Worth, TX, United States) and a non-contact wide-angle viewing system (the RESIGHT 700, Carl Zeiss Meditec AG, Jena, Germany). The vitrectomy was conducted using a cut rate of 5,000 cuts per minutes (cpm) with proportional vacuum settings and a maximum vacuum of 450 mmHg. Phacoemulsification and intraocular lens (IOL) implantation were performed simultaneously if lens opacification was visually significant.

After removing the core vitreous, the manual posterior vitreous detachment (if absent) was induced using vitreous cutter near the optic disc. Careful inspection was performed routinely with the assistance of triamcinolone and soft tip of the flute needle to detect vitreoschisis. The peripheral vitreous base was shaved up to ora serrata under 360° scleral indentation. All visible PVR membranes were peeled cautiously. After complete vitrectomy and meticulously searching for all existing breaks, the retina was flatten using fluid-air exchange (air pressure = 30 mmHg). The subretinal fluid (SRF) was drained out through existing breaks or drainage retinotomy. Retinal breaks and degenerative areas were treated by endolaser photocoagulation. Prophylactic 360° laser cerclage, perfluorocarbon liquid (PFCL), or cryopexy was not used in any eyes. At the end, the residual fluid was drained off with the flute needle to ensure complete air-fill of the vitreous cavity. Sclerotomies were carefully closed in case of air leakage.

Patients were required to maintain an alternative supine or lateral position at least 12 h/day for no less than 5 days. Follow-up examinations were scheduled at 1 day, 1 and 2 weeks, and 1, 3, and 6 months after surgery. Two weeks after surgery, axial length (AL) measurement for the highly myopic eyes was performed using Lenstar LS 900 (Haag-Streit AG, Koeniz, Switzerland, software version 1.1). If necessary, extra visits were scheduled. We prescribed dexamethasone eyedrops and ophthalmic ointment (TobraDex, Alcon) for each patient after the surgery for 2 weeks. Primary and final anatomical success rates and postoperative complications were the main outcome measures.

### Statistical Analysis

For statistical analysis, BCVA in the Snellen value was converted to the logarithm of the minimum angle of resolution (logMAR). Visual acuity of light perception, hand movements, and counting fingers were assigned as 2.9, 2.6, and 2.3, respectively (15). Mann-Whitney *U* test was employed to compare BCVA. All continuous data were expressed as mean ± standard deviation. *P* values < 0.05 were considered significant. Analyses were performed using SPSS for windows 21.0 (SPSS Inc., Chicago, IL, United States).

## Results

### Baseline Characteristics

Fifty-two eyes of 52 consecutive patients (39 men and 13 women) with causative inferior breaks were recruited. Baseline characteristics are summarized in Table 1. Supplementary Material showed the clinical characteristics and treatment outcomes of each patient. The mean age was 51.8 ± 11.8 years (range: 28–79), and the mean follow-up was 8.2 ± 1.6 months (range: 6–13 months). Sixteen patients (30.8%) presented with high myopia, with a mean AL of 28.21 ± 1.66 mm (range: 26.15–31.12, measured 2 weeks after surgery). There were 46 phakic eyes (88.5%) and six pseudophakic eyes (11.5%). The posterior lens capsule was intact in three eyes (50.0%). Yttrium–aluminum–garnet laser capsulotomy was performed in the other three eyes (50.0%). PVR grade was C1 in four eyes (7.7%), with the numeral one referring to the number of quadrants with visible PVR membrane formation.

**TABLE 1:** Clinical characteristics of 52 eyes undergone 25-G PPV with air tamponade for RRD with inferior breaks.

Characteristic	
Gender (male: female)	39 (75.0%): 13 (25.0%)
Age (years)	51.8 ± 11.8 (range: 28–79)
Disease course (days)	15.2 ± 15.1 (median:10, range: 2–90)
Preoperative logMAR BCVA (Snellen equivalent)	1.59 ± 0.93 (20/778)
Lens status (phakic: pseudophakic)	46 (88.5%): 6 (11.5%)
Vitreous status (transparent: haze: hemorrhage)	20 (38.5%): 22 (42.3%): 10 (19.2%)
High myopia (eyes)	16 (30.8%)
Axial length of highly myopic eyes (mm)	28.21 ± 1.66 (range:26.15–31.12)
PVR grade (A or none: B: C1)	22 (42.3%): 26 (50.0%): 4 (7.7%)
RRD extent (clock hours)	6.5 ± 2.3 (range: 2–12)
Macular involved (on: off)	12 (23.1%): 40 (76.9%)
Drainage retinotomy (no: yes)	25 (48.1%): 27 (51.9%)

*PPV, pars plana vitrectomy; RRD, rhegmatogenous retinal detachment; LogMAR, the logarithm of the minimum angle of resolution; BCVA, best corrected visual acuity; PVR, proliferative vitreous retinopathy.*

This series included forty-nine primary RRDs (94.2%) and three recurrent RRDs (5.8%). Of the three eyes, two were vitrectomized eyes (Case 20 underwent lens extraction and intraocular lens (IOL) implantation and silicone oil removal 7 years ago; Case 24 underwent lens extraction and IOL implantation and silicone oil removal 8 months ago). Yttrium–aluminum–garnet laser capsulotomy was performed for both patients. Another patient (Case 25) underwent scleral buckling for primary detachment 1 year ago.

Intraoperatively, we found the mean detachment extent was 6.5 ± 2.3 clock hours (range: 2–12). There was a total of 150 breaks, of which 76 (50.7%) were located in the superior retina (between 8 and 4 o’clock meridian, including the 8 and 4 o’clock meridian), and the other 74 (49.3%) were located in the inferior retina (between 4 and 8 o’clock meridian). One hundred and one breaks (67.3%) were horseshoe tears, and forty-nine breaks (32.7%) were atrophic holes. Thirteen eyes (25.0%) had inferior breaks only, and thirty-nine eyes (75.0%) had both superior and inferior breaks. Table 2 illustrates the characteristics of retinal breaks. Two eyes (3.8%) were complicated with choroidal detachment. Four patients who had lens opacification with visual significance underwent PPV combined with cataract surgery.

**TABLE 2:** Clinical characteristics of retinal breaks.

Characteristic	
**No. of breaks**	
Mean no.	2.9 ± 2.1 (range: 1–12)
Single: two: three or more (eyes)	9 (17.3%): 18 (34.6%): 25 (48.1%)
**Type of breaks**	
Horseshoe tears	29 (55.8%)
Atrophic holes	8 (15.4%)
Horseshoe tears + atrophic holes	15 (28.8%)
**Size of breaks**	
Small breaks (≤2 PD)	38 (73.1%)
Medium breaks (>2 PD, and ≤5 PD)	14 (26.9%)
**Location of breaks**	
Inferior	13 (25.0%)
Superior + inferior	39 (75.0%)

### Anatomical Outcome

Retinal reattachment after single surgery was achieved in 50 of the 52 patients (96.2%). The other two patients received vitrectomy with silicone oil tamponade as a salvage treatment, and no one had redetachment after silicone oil removal until the last follow-up. Table 3 shows the clinical characteristics of the two patients. Both eyes were attributed to new breaks, and Case 49 was related to the newly developed macular hole.

**TABLE 3:** Clinical characteristics of patients with primary reattachment failure.

Characteristics	Case 35	Case 49
Gender	Male	Male
Age (years)	49	44
Disease course (days)	10	4
Myopia	Yes	High myopia (AL: 29.55 mm)
**Preoperatively**		
Number of quadrants involved	5	6
Number of retinal breaks	4	3
Location of retinal breaks	Two in 7 o’ clock, one in 8 o’ clock, and one in 9 o’ clock	One in 7 o’ clock, one in 8 o’ clock, and one in 9 o’ clock
Type of retinal breaks	Horseshoe tears	Horseshoe tears and atrophic hole
Size of retinal breaks (PD)	1	1
Macular involved	Off	Off
PVR	None or A	None or A
Final success	Yes	Yes

### Visual Acuity Outcome

Mean preoperative logMAR BCVA (Snellen equivalent 20/796) was 1.60 ± 0.93 (range: 0–2.6, median: 1.7). The mean postoperative logMAR BCVA (Snellen equivalent 20/55) was 0.44 ± 0.24 (range: 0–1.1, median: 0.45), showing a significant improvement (*P* < 0.001).

### Complications

The air was completely absorbed 9–12 days following surgery. Temporary IOP elevation (>21 mmHg) occurred in seven highly myopic patients 1 week after the surgery and was controlled well using topical medications without any permanent damage. Ocular hypotony, endophthalmitis, and other serious complications were not observed. During follow-up, eight eyes (17.4%) of the 46 phakic eyes underwent secondary cataract surgery.

## Discussion

This retrospective consecutive case series included fifty-two eyes of fifty-two RRD patients, demonstrating that inferior breaks in RRDs can be effectively managed with 25-G PPV combined with air tamponade. After a follow-up of at least 6 months, fifty patients (96.2%) achieved successful retinal reattachment after a single surgery, with corresponding improvements in BCVA. This technique demonstrated favorable surgical outcomes and fewer complications.

Conventionally, long-term tamponades, such as C_3_F_8_ and silicone oil, are commonly used. However, long-acting gas significantly affects postoperative visual rehabilitation and requires a prolonged prone position. Silicone oil can also lead to many potential complications, such as secondary glaucoma and cataract. Moreover, these patients require additional surgery to remove silicone oil. Therefore, attempts have been made to shorten the prone positioning period and minimize complications by using air.

Using air tamponade has several important advantages. Air, with a much shorter half-life and the non-expansile property, usually remains in the eye for less than 1 week (16), allows much quicker visual rehabilitation, and reduces postoperative complications, such as elevated IOP and PVR (11, 17). Like the long-acting gas, air provides buoyant force and great surface tension to seal retinal breaks, preventing fluid accumulation in the subretinal space. A previous study showed that the retina-RPE adhesion occurs within 24 h in situations without SRF (18). After that time, fluid will not enter the subretinal space through breaks. Therefore, the long-term tamponade may be unnecessary for retinal detachment. Moreover, the shorter duration of air in the eye may mitigate against postoperative PVR or epiretinal membrane formation (19).

The locations of retinal breaks and quadrants involved are important and clinically relevant factors that may affect surgical outcomes (5). Inferior breaks always pose a challenge to intraocular air tamponade. Previous studies showed conflicting results. Tan et al. (10) found that RRDs that involved the inferior quadrants had significantly lower primary success rates when using air tamponade, compared to SF_6_ tamponade. They suggested that air tamponade should only be used in superior RRDs. Nevertheless, in their study, shaving of the vitreous base was only performed around retinal breaks, and cryocoagulation was used in all cases, which is known to require longer time to induce chorioretinal adhesion than laser coagulation. A prospective randomized study by Zhou et al. (11) indicated that air had equivalent tamponade effects to C_3_F_8_ for RRDs with inferior breaks (single-operation success rate: 84 and 78% in air and C_3_F_8_ groups, respectively). Martínez-Castillo et al. (12, 13) reported that PPV with air tamponade was effective in the treatment of pseudophakic RRDs with inferior breaks without facedown position postoperatively (primary success rate: 90–93.3%). Consistent with previously reported results, this case series, with single and final operation success rates of 96.2 and 100%, respectively, provides further evidence that air tamponade is sufficient to establish a stable chorioretinal adhesion and achieve retinal reattachment in RRDs with inferior breaks.

For RRDs with inferior breaks, it may be challenging to maintain the retinal breaks attached until the chorioretinal adhesion developed. For RRDs with superior breaks, air in the vitreous cavity can seal the breaks easily because of buoyant force and great surface tension. Regarding RRDs with inferior breaks, air may be unlikely to provide effective tamponade for the breaks for a sufficient duration postoperatively, as the residual SRF tends to fall to the inferior quadrant and may seep around edge of the inferior breaks due to gravity. Thus, it is assumed that air tamponade may be insufficient for the treatment of RRDs with inferior breaks. However, Martínez-Castillo et al. (12, 20) reported that 20-G PPV with air tamponade could adequately treat RRDs with inferior breaks, if complete drainage of SRF was performed. Tetsumoto et al. (9) inferred that short-term tamponade may sufficiently reduce the risk of redetachment if the SRF does not reach the original break. In present study, we adjusted the head position appropriately during the fluid-air exchange, allowing the SRF to flow out easily. In patients with a tiny break in the far periphery, a drainage retinotomy can be created to fully drain the SRF. A peripheral drainage retinotomy should be attempted in a superior quadrant, whenever possible, to avoid complications. Moreover, postoperative supine positioning could prevent the SRF from collecting around the inferior breaks, giving sufficient duration for the establishment of stable chorioretinal adhesion. A recent study by Gozawa et al. (21) observed intraocular gas contact rates of the retina using MRI. They found that the gas could adequately support and seal superior and inferior parts of the retina in the supine position, with a gas contact rate exceeding 90%. Therefore, the patients in this study were instructed to maintain an alternative supine or lateral position, implying that intraocular air bubble can effectively seal inferior breaks and enhance SRF absorption. In addition, the supine position is less demanding and easier for the patients to maintain.

Previous literature demonstrated that the primary anatomical success rates of air tamponade varied from 73 to 94.5% (11, 12, 20, 22–24). This discrepancy may be due to different patient selection criteria, varied sample sizes, and diverse vitreoretinal surgical techniques. It should be noted that surgical techniques are essential for a successful RRD repair. The pathogenesis of RRD involves vitreoretinal tractional forces that result in a full-thickness break (1). Therefore, our technique highlights complete vitreous removal. The vitreous around retinal breaks and vitreous base were removed as completely as possible under 360° scleral indentation. A wide-angle viewing system and scleral indentation are critical for a successful shaving of the vitreous base. In addition, advanced vitreous cutter and directional endolaser probe have greatly facilitated the management of retinal breaks in peripheral, reducing complications such as iatrogenic breaks and posterior capsular damage. Triamcinolone was also used routinely to detect the residual vitreous gel in case of insufficient vitreous liquefaction. In addition, excessive interventions should be avoided to prevent unnecessary complications. PFCL is usually used during vitrectomy to facilitate the peripheral vitreous shaving and SRF drainage. However, subretinal migration of PFCL is a common complication. Li et al. (5) reported that a 360° prophylactic laser coagulation could improve anatomical success rate. It is essential to identify and treat all retinal breaks. Nevertheless, excessive laser may cause retinal necrosis and small, difficult-to-find retinal holes, leading to a redetachment. These holes are difficult to identify within the patches of chorioretinal atrophy. In present study, most patients had severe vitreous liquefaction or horseshoe tears with strong vitreoretinal traction, or a combination of these factors. Therefore, we did not choose scleral buckling as the primary treatment. Our experience with this case series provides further evidence that the complete vitrectomy with air tamponade could be effective in repairing RRDs with inferior breaks. After a follow-up of at least 6 months, the retina of fifty eyes completely reattached after a single surgery, obtaining a primary success rate of 96.2%.

No serious adverse events occurred during the procedure, which indicated the safety of the current surgical technique. As previously demonstrated (10, 11, 20), the leading cause of redetachment is new breaks, which occurred in both recurrent patients. One patient (Case 49) complicated with high myopia developed a myopic macular hole-associated retinal detachments 2 weeks after surgery. As a small amount of submacular fluid would be left at the end of surgery, a potential explanation may be that the great surface tension of air presses against the fovea and the submacular fluid. Previous literature disclosed that exposure of the lens to abnormally high oxygen levels can lead to nuclear sclerosis in vitrectomized eyes (25). In this case series, eight eyes (17.4%) underwent secondary cataract surgery during follow-up. The air usually remains in the eye for about 1 week. The shorter duration of intraocular air contacting with the lens may reduce the risk of cataract development or progression. This study has several limitations, including its retrospective design and the lack of a control group. The retrospective design has an inherent risk of selection bias. In addition, all patients were enrolled from a single tertiary institution, which may cause selection bias.

In conclusion, we observed a satisfactory success rate using 25-G PPV with air tamponade in repairing RRDs with inferior breaks. This technique has a faster visual rehabilitation, a shorter positioning period, fewer complications, and reduced medical costs. The high success rate of this study suggests that air has emerged as a reasonable alternative for tamponade in the management of RRDs with inferior breaks. Prospective comparative studies to assess the efficacy of this technique for complex RRDs are required in the future work.

### Typical Case Presentation

**FIGURE 1:**
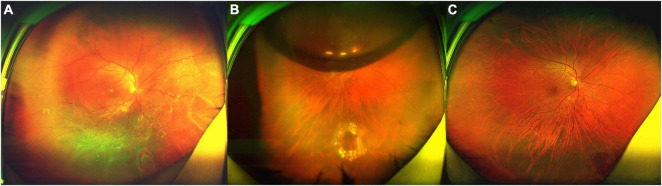
Fundus photograph taken preoperatively and postoperatively of the patient (No. 15) undergoing 25-G PPV with air tamponade. **(A)** A total RRD (range: 12 clock hours) with an inferonasal horseshoe tear (2.5 PD) and posterior PVR. **(B)** Postoperative 6-day follow-up: the retina reattached. There was an air bubble in the vitreous cavity. **(C)** Postoperative 6-month follow-up: the retina reattached, with the firm chorioretinal adhesion induced by laser coagulation.

Patient No. 15 (Figure 1).

A 46-year-old highly myopic man was referred to our clinic. He complained of decreased vision in the right eye, which had persisted for about 40 days prior to his initial visit to our clinic. His BCVA was 1.7 logMAR (Snellen equivalent 20/1,000). The AL was 26.29 mm in the right eye, which was measured by Lenstar 2 weeks after surgery. Fundoscopy revealed a total RRD (range: 12 clock hours) with posterior PVR and an inferonasal horseshoe tear (2.5 PD) (Figure 1A). Six days after surgery, the retina reattached (Figure 1B). Six months after surgery, the BCVA in right eye was 0.6 logMAR (Snellen equivalent 20/80, Figure 1C).

**FIGURE 2:**
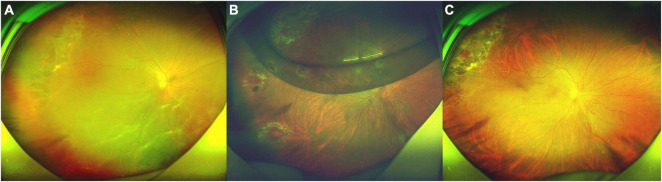
Fundus photograph taken preoperatively and postoperatively of the patient (No. 27) undergoing 25-G PPV with air tamponade. **(A)** A macular-off RRD (range: 11 clock hours) with nine horseshoe tears in superotemporal, inferotemporal, and inferonasal quadrants and posterior PVR. **(B)** Postoperative 5-day follow-up: the retina reattached. There was an air bubble in the vitreous cavity. **(C)** Postoperative 4-month follow-up: the retina reattached, with the firm chorioretinal adhesion induced by laser coagulation.

Patient No. 27 (Figure 2).

A 61-year-old man presented with darkness and decreased vision in the right eye that developed over 1 month. He was previously treated by laser retinopexy for superotemporal tears and localized detachment 20 days ago, but developed a macular-off retinal detachment with nine horseshoe tears and posterior PVR. Intraoperatively, he was found to have multiple horseshoe tears along the attachment of the posterior hyaloid to the posterior vitreous base. His BCVA was 1.7 logMAR (Snellen equivalent 20/1000). Fundoscopy revealed a macular-off RRD (range: 11 clock hours) with nine horseshoe tears (Figure 2A). Five days after surgery, the retina reattached (Figure 2B). Four months after surgery, the BCVA in right eye was 0.6 logMAR (Snellen equivalent 20/80, Figure 2C).

## Data Availability Statement

The data can be obtained with a request to the corresponding authors. There is no confidential data or any restriction on accession to the original data.

## Ethics Statement

The studies involving human participants were reviewed and approved by Ethics Committee of the Second People’s Hospital of Foshan. The patients/participants provided their written informed consent to participate in this study. Written informed consent was obtained from the individual(s) for the publication of any potentially identifiable images or data included in this article.

## Author Contributions

PSh and XK designed the study. PSh, XK, and XL had full access to all data in the study and take responsibility for the integrity of the data and the accuracy of the data analysis. PSh, XK, LZ, and PSu had roles in the clinical management, patient recruitment, and clinical data collection. PSh had roles in data collection and statistical analysis. PSh wrote the manuscript. XL and MH contributed to critical revision of the report. All authors reviewed and approved the final version of the manuscript.

## Conflict of Interest

The authors declare that the research was conducted in the absence of any commercial or financial relationships that could be construed as a potential conflict of interest.

## Publisher’s Note

All claims expressed in this article are solely those of the authors and do not necessarily represent those of their affiliated organizations, or those of the publisher, the editors and the reviewers. Any product that may be evaluated in this article, or claim that may be made by its manufacturer, is not guaranteed or endorsed by the publisher.
