# Supplementary material for: Air Tamponade for Rhegmatogenous Retinal Detachment With Inferior Breaks After 25-Gauge Pars Plana Vitrectomy: Technique and Outcome
Source: Front Med (Lausanne). 2022 Apr 7;9:724234. doi: 10.3389/fmed.2022.724234 (PMC9021743; doi:10.3389/fmed.2022.724234)
Supplement: Supplementary file 1 [file Table_1.docx]

**Supplementary Table**. Characteristics and Visual Outcome of 52 Patients Undergone PPV with Air Tamponade for RRD with Inferior Breaks

| No. | Age/Sex | Eye affected | Disease course (days) | Lens status | Vitreous status | High myopia status (Axial length) | Detachment extent (clock hours) | Macular involved | PVR grading | Number of breaks | Cataract surgery | Drainage retinotomy | LogMAR BCVA (Snellen equivalent) | | |
| --- | --- | --- | --- | --- | --- | --- | --- | --- | --- | --- | --- | --- | --- | --- | --- |
|  |  |  |  |  |  |  |  |  |  |  |  |  | preoperative | postoperative |  |
| 1 | 58/M | OS | 21 | Pseudophakic | Haze | No | 6 | On | B | 2 | No | Yes | 0 (20/20) | 0 (20/20) |  |
| 2 | 42/M | OS | 14 | Phakic | Haze | Yes (26.58 mm) | 5 | Off | B | 4 | No | Yes | 1.1 (20/250) | 0.2 (20/32) |  |
| 3 | 50/F | OD | 30 | Phakic | Hemorrhage | No | 6 | On | B | 3 | No | Yes | 0.5 (20/63) | 0.1 (20/25) |  |
| 4 | 59/M | OS | 30 | Phakic | Hemorrhage | No | 6 | Off | B | 1 | No | No | 1.3 (20/400) | 0.2 (20/32) |  |
| 5 | 52/F | OD | 15 | Phakic | Transparent | No | 2 | Off | A | 2 | No | Yes | 1.0 (20/200) | 0.1 (20/25) |  |
| 6 | 63/M | OD | 3 | Phakic | Transparent | No | 3 | On | A | 4 | No | No | 0 (20/20) | 0 (20/20) |  |
| 7 | 42/M | OS | 2 | Phakic | Transparent | Yes (29.91 mm) | 8 | Off | A | 3 | No | Yes | 2.3 (20/2000) | 1.1 (20/250) |  |
| 8 | 36/M | OS | 2 | Phakic | Transparent | Yes (29.12 mm) | 4 | Off | A | 2 | No | No | 2.3 (20/2000) | 0.8 (20/125) |  |
| 9 | 61/M | OD | 5 | Phakic | Transparent | No | 5 | Off | B | 3 | No | No | 2.6 (20/20000) | 0.4 (20/50) |  |
| 10 | 57/M | OD | 10 | Phakic | Transparent | No | 7 | Off | B | 2 | No | No | 2.3 (20/2000) | 0.6 (20/80) |  |
| 11 | 49/M | OS | 7 | Phakic | Transparent | Yes (28.56 mm) | 6 | Off | A | 2 | No | No | 1.7 (20/1000) | 0.5 (20/63) |  |
| 12 | 52/F | OS | 3 | Pseudophakic | Hemorrhage | No | 2 | On | A | 1 | No | No | 0.2 (20/32) | 0.1 (20/25) |  |
| 13 | 45/F | OD | 30 | Phakic | Hemorrhage | No | 7 | Off | B | 2 | No | No | 2.6 (20/20000) | 0.5 (20/63)) |  |
| 14 | 65/F | OS | 20 | Phakic | Haze | No | 8 | Off | B | 3 | No | Yes | 2.3 (20/2000) | 0.7 (20/100) |  |
| 15 | 46/M | OD | 40 | Phakic | Transparent | Yes (26.29 mm) | 12 | Off | C1 | 1 | No | No | 1.7 (20/1000) | 0.6 (20/80) |  |
| 16 | 63/F | OD | 30 | Phakic | Haze | No | 8 | Off | B | 2 | No | Yes | 2.3 (20/2000) | 0.6 (20/80) |  |
| 17 | 30/M | OS | 14 | Phakic | Haze | Yes (31.12 mm) | 7 | Off | B | 2 | No | Yes | 2.6 (20/20000) | 0.5 (20/63) |  |
| 18 | 74/M | OD | 14 | Phakic | Haze | No | 6 | Off | B | 1 | No | No | 2.6 (20/20000) | 0.4 (20/50) |  |
| 19 | 64/F | OD | 90 | Phakic | Transparent | No | 6 | On | B | 1 | No | No | 1.0 (20/200) | 0.6 (20/80) |  |
| 20 | 28/F | OD | 5 | Pseudophakic | Haze | Yes (26.75 mm) | 6 | On | A | 3 | No | No | 0.7 (20/100) | 0.6 (20/80) |  |
| 21 | 50/M | OD | 12 | Phakic | Hemorrhage | No | 9 | Off | B | 3 | No | No | 2.6 (20/20000) | 0.5 (20/63) |  |
| 22 | 51/M | OS | 7 | Phakic | Transparent | Yes (27.86 mm) | 6 | Off | B | 2 | No | Yes | 1.7 (20/1000) | 0.4 (20/50) |  |
| 23 | 55/F | OD | 7 | Phakic | Hemorrhage | No | 5 | Off | B | 4 | No | Yes | 2.6 (20/20000) | 0.5 (20/63) |  |
| 24 | 51/M | OS | 3 | Pseudophakic | Transparent | No | 5 | Off | A | 2 | No | Yes | 1.7 (20/1000) | 0.7 (20/100) |  |
| 25 | 46/M | OD | 7 | Phakic | Haze | Yes (27.15 mm) | 3 | On | B | 1 | No | Yes | 0.1 (20/25) | 0.1 (20/25) |  |
| 26 | 78/M | OD | 30 | Pseudophakic | Haze | No | 6 | Off | B | 1 | No | No | 2.6 (20/20000) | 0.6 (20/80) |  |
| 27 | 61/M | OD | 30 | Phakic | Haze | No | 11 | Off | C1 | 9 | No | Yes | 1.7 (20/1000) | 0.6 (20/80) |  |
| 28 | 64/M | OD | 3 | Phakic | Transparent | No | 5 | Off | A | 3 | No | Yes | 1.2 (20/320) | 0.4 (20/50) |  |
| 29 | 66/M | OS | 25 | Phakic | Haze | No | 6 | Off | C1 | 4 | Yes | Yes | 2.3 (20/2000) | 0.7 (20/100) |  |
| 30 | 34/M | OD | 9 | Phakic | Hemorrhage | Yes (26.25 mm) | 4 | On | A | 2 | No | Yes | 0 (20/20) | 0 (20/20) |  |
| 31 | 44/M | OD | 10 | Pseudophakic | Haze | Yes (26.15 mm) | 6 | On | A | 1 | No | No | 0.2 (20/32) | 0 (20/20) |  |
| 32 | 35/M | OD | 35 | Phakic | Haze | No | 10 | Off | B | 3 | No | No | 2.6 (20/20000) | 0.6 (20/80) |  |
| 33 | 59/M | OD | 8 | Phakic | Haze | No | 7 | Off | B | 2 | No | No | 1.7 (20/1000) | 0.7 (20/100) |  |
| 34 | 42/M | OD | 2 | Phakic | Hemorrhage | No | 11 | Off | A | 12 | No | Yes | 2.6 (20/20000) | 0.5 (20/63) |  |
| 35 | 49/M | OD | 10 | Phakic | Transparent | No | 5 | Off | A | 4 | No | No | 1.0 (20/200) | 0.6 (20/80) |  |
| 36 | 50/M | OD | 30 | Phakic | Transparent | No | 6 | Off | A | 2 | No | Yes | 0.7 (20/100) | 0.5 (20/63) |  |
| 37 | 43/F | OD | 20 | Phakic | Haze | No | 8 | Off | A | 3 | No | Yes | 1.2 (20/320) | 0.2 (20/32) |  |
| 38 | 43/M | OD | 6 | Phakic | Transparent | No | 10 | Off | A | 3 | No | Yes | 0.8 (20/125) | 0.4 (20/50) |  |
| 39 | 58/M | OS | 15 | Phakic | Haze | No | 12 | Off | A | 3 | No | No | 2.6 (20/20000) | 0.6 (20/80) |  |
| 40 | 38/F | OD | 14 | Phakic | Haze | Yes (27.35 mm) | 8 | Off | A | 9 | No | Yes | 0.8 (20/125) | 0.4 (20/50) |  |
| 41 | 59/M | OD | 14 | Phakic | Transparent | No | 6 | On | A | 2 | No | Yes | 0.1 (20/25) | 0.3 (20/40) |  |
| 42 | 79/F | OD | 6 | Phakic | Transparent | No | 6 | Off | A | 2 | Yes | Yes | 2.3 (20/2000) | 0.7 (20/100) |  |
| 43 | 63/F | OS | 30 | Phakic | Haze | No | 8 | Off | B | 3 | No | No | 0.6 (20/80) | 0 (20/20) |  |
| 44 | 58/M | OD | 4 | Phakic | Haze | No | 9 | Off | A | 4 | No | Yes | 1.0 (20/200) | 0.4 (20/50) |  |
| 45 | 33/M | OD | 4 | Phakic | Hemorrhage | Yes (30.88 mm) | 7 | Off | A | 2 | No | No | 2.6 (20/20000) | 0.7 (20/100) |  |
| 46 | 38/M | OS | 4 | Phakic | Haze | Yes (28.50 mm) | 7 | Off | A | 4 | No | No | 2.3 (20/2000) | 0.6 (20/80) |  |
| 47 | 67/M | OS | 3 | Phakic | Transparent | No | 4 | Off | A | 2 | Yes | Yes | 2.6 (20/20000) | 0.5 (20/63) |  |
| 48 | 51/M | OD | 3 | Phakic | Haze | No | 5 | On | A | 3 | No | Yes | 0 (20/20) | 0.3 (20/40) |  |
| 49 | 44/M | OD | 4 | Phakic | Transparent | Yes (29.55 mm) | 6 | Off | A | 3 | Yes | No | 2.6 (20/20000) | 0.8 (20/125) |  |
| 50 | 45/M | OD | 30 | Phakic | Hemorrhage | Yes (29.40 mm) | 4 | On | A | 1 | No | No | 2.3 (20/2000) | 0.6 (20/80) |  |
| 51 | 57/M | OD | 15 | Phakic | Transparent | No | 7 | Off | A | 2 | No | No | 2.6 (20/20000) | 0.4 (20/50) |  |
| 52 | 48/M | OD | 3 | Phakic | Haze | No | 7 | Off | A | 5 | No | Yes | 1.7 (20/1000) | 0.4 (20/50) |  |

PPV, pars plana vitrectomy; RRD, rhegmatogenous retinal detachment；LogMAR, the logarithm of the minimum angle of resolution; BCVA, best corrected visual acuity; PVR, proliferative vitreous retinopathy.
